# Supplementary material for: Measurement and spatiotemporal analysis of high-quality development of China’s industry
Source: PLoS One. 2021 Dec 31;16(12):e0259845. doi: 10.1371/journal.pone.0259845 (PMC8719736; doi:10.1371/journal.pone.0259845)
Supplement: S1 Table — (DOCX) [file pone.0259845.s001.docx]

**Table.** Relative nearness degree and rank of 30 provinces from 1999 to 2018.

|  | 1999 | | 2000 | | 2001 | | 2002 | | 2003 | |
| --- | --- | --- | --- | --- | --- | --- | --- | --- | --- | --- |
| Province | ND | Rank | ND | Rank | ND | Rank | ND | Rank | ND | Rank |
| Beijing | 0.4799 | 3 | 0.4492 | 3 | 0.4095 | 7 | 0.4164 | 7 | 0.4413 | 7 |
| Tianjin | 0.3815 | 7 | 0.3396 | 7 | 0.4955 | 5 | 0.5207 | 4 | 0.5671 | 4 |
| Hebei | 0.1864 | 22 | 0.1401 | 26 | 0.1919 | 24 | 0.2137 | 20 | 0.2164 | 21 |
| Shanxi | 0.1170 | 27 | 0.1111 | 29 | 0.1757 | 25 | 0.1818 | 24 | 0.2089 | 22 |
| Inner Mongolia | 0.1776 | 23 | 0.1610 | 23 | 0.2700 | 13 | 0.1779 | 26 | 0.1850 | 26 |
| Liaoning | 0.2811 | 15 | 0.2053 | 18 | 0.2783 | 12 | 0.2998 | 10 | 0.3347 | 9 |
| Jilin | 0.1997 | 21 | 0.1909 | 21 | 0.2013 | 23 | 0.2041 | 21 | 0.2030 | 23 |
| Heilongjiang | 0.2871 | 14 | 0.4032 | 4 | 0.3590 | 8 | 0.3972 | 8 | 0.3420 | 8 |
| Shanghai | 0.4121 | 6 | 0.3749 | 5 | 0.5374 | 2 | 0.5422 | 2 | 0.6240 | 2 |
| Jiangsu | 0.3205 | 10 | 0.3095 | 9 | 0.4264 | 6 | 0.4335 | 6 | 0.4869 | 6 |
| Zhejiang | 0.4233 | 4 | 0.3529 | 6 | 0.5152 | 3 | 0.5259 | 3 | 0.4900 | 5 |
| Anhui | 0.3151 | 11 | 0.2836 | 12 | 0.2302 | 16 | 0.2278 | 19 | 0.2367 | 20 |
| Fujian | 0.5414 | 1 | 0.5844 | 1 | 0.5001 | 4 | 0.4910 | 5 | 0.5986 | 3 |
| Jiangxi | 0.2512 | 17 | 0.2252 | 16 | 0.1643 | 28 | 0.1811 | 25 | 0.1996 | 24 |
| Shandong | 0.3303 | 9 | 0.3110 | 8 | 0.3164 | 9 | 0.3044 | 9 | 0.3237 | 10 |
| Henan | 0.2675 | 16 | 0.2190 | 17 | 0.1524 | 30 | 0.1652 | 28 | 0.1708 | 28 |
| Hubei | 0.3107 | 12 | 0.2833 | 13 | 0.2187 | 20 | 0.2362 | 17 | 0.2389 | 19 |
| Hunan | 0.2920 | 13 | 0.2716 | 14 | 0.2281 | 18 | 0.2486 | 12 | 0.2502 | 14 |
| Guangdong | 0.5072 | 2 | 0.4963 | 2 | 0.5665 | 1 | 0.5920 | 1 | 0.6316 | 1 |
| Guangxi | 0.2175 | 20 | 0.2047 | 19 | 0.2094 | 22 | 0.2039 | 22 | 0.2420 | 18 |
| Hainan | 0.3385 | 8 | 0.2957 | 10 | 0.2927 | 11 | 0.2454 | 14 | 0.2932 | 11 |
| Chongqing | 0.4233 | 5 | 0.1963 | 20 | 0.2295 | 17 | 0.2426 | 15 | 0.2742 | 12 |
| Sichuan | 0.1714 | 24 | 0.1488 | 24 | 0.2121 | 21 | 0.2361 | 18 | 0.2449 | 17 |
| Guizhou | 0.1157 | 28 | 0.1149 | 28 | 0.1646 | 27 | 0.1711 | 27 | 0.1751 | 27 |
| Yunnan | 0.2478 | 18 | 0.2858 | 11 | 0.3026 | 10 | 0.2409 | 16 | 0.2451 | 16 |
| Shaanxi | 0.1687 | 25 | 0.1645 | 22 | 0.2376 | 15 | 0.2637 | 11 | 0.2651 | 13 |
| Gansu | 0.0865 | 30 | 0.0843 | 30 | 0.1525 | 29 | 0.1349 | 30 | 0.1692 | 29 |
| Qinghai | 0.1147 | 29 | 0.1258 | 27 | 0.2224 | 19 | 0.1385 | 29 | 0.1484 | 30 |
| Ningxia | 0.1566 | 26 | 0.1426 | 25 | 0.1688 | 26 | 0.1901 | 23 | 0.1917 | 25 |
| Xinjiang | 0.2192 | 19 | 0.2337 | 15 | 0.2660 | 14 | 0.2483 | 13 | 0.2500 | 15 |

**Table.** Relative nearness degree and rank of 30 provinces from 1999 to 2018.

|  | 2004 | | 2005 | | 2006 | | 2007 | | 2008 | |
| --- | --- | --- | --- | --- | --- | --- | --- | --- | --- | --- |
| Province | ND | Rank | ND | Rank | ND | Rank | ND | Rank | ND | Rank |
| Beijing | 0.4106 | 6 | 0.3855 | 8 | 0.3634 | 6 | 0.3801 | 2 | 0.4957 | 2 |
| Tianjin | 0.5531 | 3 | 0.5337 | 2 | 0.3804 | 4 | 0.3095 | 4 | 0.4630 | 5 |
| Hebei | 0.1748 | 24 | 0.1668 | 25 | 0.1436 | 24 | 0.1191 | 17 | 0.1503 | 26 |
| Shanxi | 0.1867 | 20 | 0.1707 | 24 | 0.1514 | 22 | 0.0874 | 26 | 0.1426 | 28 |
| Inner Mongolia | 0.1649 | 26 | 0.1573 | 26 | 0.1415 | 26 | 0.0953 | 24 | 0.1555 | 24 |
| Liaoning | 0.2912 | 10 | 0.2411 | 12 | 0.2010 | 14 | 0.1335 | 14 | 0.2150 | 13 |
| Jilin | 0.1878 | 19 | 0.1830 | 23 | 0.1559 | 21 | 0.1226 | 15 | 0.1703 | 22 |
| Heilongjiang | 0.3397 | 9 | 0.3546 | 9 | 0.2777 | 10 | 0.1628 | 11 | 0.2639 | 9 |
| Shanghai | 0.6135 | 1 | 0.5354 | 1 | 0.4198 | 3 | 0.3392 | 3 | 0.5093 | 1 |
| Jiangsu | 0.4636 | 5 | 0.4234 | 6 | 0.3734 | 5 | 0.2276 | 7 | 0.4199 | 6 |
| Zhejiang | 0.3936 | 7 | 0.3887 | 7 | 0.3303 | 8 | 0.1858 | 9 | 0.3326 | 8 |
| Anhui | 0.1966 | 18 | 0.1894 | 21 | 0.1476 | 23 | 0.1103 | 20 | 0.2135 | 15 |
| Fujian | 0.4727 | 4 | 0.4416 | 5 | 0.3474 | 7 | 0.2143 | 8 | 0.3455 | 7 |
| Jiangxi | 0.1606 | 28 | 0.2192 | 15 | 0.1739 | 16 | 0.0910 | 25 | 0.1829 | 19 |
| Shandong | 0.2776 | 12 | 0.2711 | 11 | 0.2393 | 12 | 0.1377 | 12 | 0.2438 | 10 |
| Henan | 0.1577 | 29 | 0.1559 | 27 | 0.1331 | 27 | 0.1170 | 19 | 0.1541 | 25 |
| Hubei | 0.2341 | 15 | 0.1963 | 19 | 0.1701 | 18 | 0.1036 | 21 | 0.1925 | 17 |
| Hunan | 0.1848 | 22 | 0.1928 | 20 | 0.1628 | 20 | 0.0997 | 23 | 0.1910 | 18 |
| Guangdong | 0.5578 | 2 | 0.4942 | 4 | 0.4232 | 2 | 0.2704 | 5 | 0.4920 | 3 |
| Guangxi | 0.1859 | 21 | 0.1886 | 22 | 0.1329 | 28 | 0.0781 | 28 | 0.1444 | 27 |
| Hainan | 0.3665 | 8 | 0.4998 | 3 | 0.5813 | 1 | 0.6884 | 1 | 0.1747 | 21 |
| Chongqing | 0.2789 | 11 | 0.2005 | 17 | 0.1659 | 19 | 0.1179 | 18 | 0.2316 | 11 |
| Sichuan | 0.1790 | 23 | 0.1984 | 18 | 0.1715 | 17 | 0.1030 | 22 | 0.1776 | 20 |
| Guizhou | 0.1637 | 27 | 0.1535 | 28 | 0.1429 | 25 | 0.0869 | 27 | 0.2117 | 16 |
| Yunnan | 0.2121 | 16 | 0.2076 | 16 | 0.2449 | 11 | 0.1757 | 10 | 0.1696 | 23 |
| Shaanxi | 0.2634 | 14 | 0.2403 | 13 | 0.1777 | 15 | 0.1202 | 16 | 0.2137 | 14 |
| Gansu | 0.1238 | 30 | 0.1391 | 29 | 0.1010 | 30 | 0.0733 | 29 | 0.1310 | 30 |
| Qinghai | 0.1975 | 17 | 0.2270 | 14 | 0.2341 | 13 | 0.1335 | 13 | 0.4830 | 4 |
| Ningxia | 0.1678 | 25 | 0.1264 | 30 | 0.1223 | 29 | 0.0692 | 30 | 0.1332 | 29 |
| Xinjiang | 0.2658 | 13 | 0.2780 | 10 | 0.2795 | 9 | 0.2329 | 6 | 0.2282 | 12 |

**Table .** Relative nearness degree and rank of 30 provinces from 1999 to 2018.

|  | 2009 | | 2010 | | 2011 | | 2012 | | 2013 | |
| --- | --- | --- | --- | --- | --- | --- | --- | --- | --- | --- |
| Province | ND | Rank | ND | Rank | ND | Rank | ND | Rank | ND | Rank |
| Beijing | 0.5368 | 3 | 0.5351 | 5 | 0.5719 | 4 | 0.5599 | 3 | 0.5784 | 2 |
| Tianjin | 0.5017 | 5 | 0.5542 | 4 | 0.5359 | 5 | 0.4838 | 5 | 0.4329 | 7 |
| Hebei | 0.1454 | 28 | 0.1896 | 25 | 0.1812 | 24 | 0.1828 | 25 | 0.1543 | 25 |
| Shanxi | 0.1403 | 29 | 0.1668 | 28 | 0.1679 | 27 | 0.1832 | 24 | 0.1734 | 22 |
| Inner Mongolia | 0.1676 | 24 | 0.1807 | 26 | 0.1659 | 28 | 0.1648 | 28 | 0.3462 | 10 |
| Liaoning | 0.2566 | 10 | 0.2762 | 12 | 0.2653 | 11 | 0.2316 | 14 | 0.2101 | 17 |
| Jilin | 0.2508 | 11 | 0.2011 | 21 | 0.1939 | 22 | 0.1892 | 22 | 0.1570 | 24 |
| Heilongjiang | 0.1963 | 21 | 0.2137 | 18 | 0.2206 | 18 | 0.2022 | 19 | 0.1538 | 26 |
| Shanghai | 0.6552 | 1 | 0.7564 | 1 | 0.7780 | 1 | 0.6663 | 1 | 0.6933 | 1 |
| Jiangsu | 0.5062 | 4 | 0.5680 | 3 | 0.5972 | 3 | 0.6658 | 2 | 0.5675 | 3 |
| Zhejiang | 0.3885 | 8 | 0.4284 | 8 | 0.4666 | 7 | 0.4639 | 6 | 0.4079 | 8 |
| Anhui | 0.2395 | 12 | 0.2110 | 19 | 0.2296 | 15 | 0.3049 | 10 | 0.2412 | 12 |
| Fujian | 0.4325 | 7 | 0.4654 | 6 | 0.4432 | 8 | 0.4189 | 8 | 0.4011 | 9 |
| Jiangxi | 0.2066 | 18 | 0.2390 | 15 | 0.2345 | 14 | 0.2119 | 18 | 0.1997 | 19 |
| Shandong | 0.2272 | 13 | 0.3030 | 10 | 0.2816 | 10 | 0.2741 | 12 | 0.2448 | 11 |
| Henan | 0.1511 | 27 | 0.1667 | 29 | 0.1805 | 26 | 0.1884 | 23 | 0.1835 | 20 |
| Hubei | 0.2158 | 16 | 0.2467 | 13 | 0.2502 | 12 | 0.2389 | 13 | 0.2106 | 16 |
| Hunan | 0.2193 | 14 | 0.3394 | 9 | 0.2255 | 16 | 0.2256 | 15 | 0.2164 | 15 |
| Guangdong | 0.5840 | 2 | 0.6495 | 2 | 0.6105 | 2 | 0.5515 | 4 | 0.5002 | 4 |
| Guangxi | 0.1681 | 23 | 0.1949 | 23 | 0.1809 | 25 | 0.1679 | 26 | 0.1519 | 27 |
| Hainan | 0.4761 | 6 | 0.4375 | 7 | 0.5094 | 6 | 0.4590 | 7 | 0.4648 | 5 |
| Chongqing | 0.2674 | 9 | 0.2834 | 11 | 0.3129 | 9 | 0.3737 | 9 | 0.4495 | 6 |
| Sichuan | 0.2079 | 17 | 0.2210 | 16 | 0.2501 | 13 | 0.2976 | 11 | 0.2269 | 14 |
| Guizhou | 0.1675 | 25 | 0.2043 | 20 | 0.1891 | 23 | 0.1655 | 27 | 0.1385 | 29 |
| Yunnan | 0.2179 | 15 | 0.2399 | 14 | 0.2167 | 19 | 0.1907 | 21 | 0.1641 | 23 |
| Shaanxi | 0.2045 | 19 | 0.2144 | 17 | 0.2097 | 20 | 0.2154 | 17 | 0.1799 | 21 |
| Gansu | 0.1622 | 26 | 0.1898 | 24 | 0.2255 | 17 | 0.2172 | 16 | 0.2388 | 13 |
| Qinghai | 0.1264 | 30 | 0.1487 | 30 | 0.1474 | 30 | 0.1344 | 30 | 0.0978 | 30 |
| Ningxia | 0.1984 | 20 | 0.1964 | 22 | 0.1959 | 21 | 0.2003 | 20 | 0.1451 | 28 |
| Xinjiang | 0.1738 | 22 | 0.1698 | 27 | 0.1524 | 29 | 0.1370 | 29 | 0.2035 | 18 |

**Table .** Relative nearness degree and rank of 30 provinces from 1999 to 2018.

|  | 2014 | | 2015 | | 2016 | | 2017 | | 2018 | |
| --- | --- | --- | --- | --- | --- | --- | --- | --- | --- | --- |
| Province | ND | Rank | ND | Rank | ND | Rank | ND | Rank | ND | Rank |
| Beijing | 0.5623 | 2 | 0.4394 | 4 | 0.4704 | 2 | 0.5429 | 2 | 0.2843 | 3 |
| Tianjin | 0.4087 | 6 | 0.3125 | 7 | 0.2929 | 8 | 0.3855 | 7 | 0.2174 | 8 |
| Hebei | 0.1463 | 27 | 0.1270 | 25 | 0.1236 | 24 | 0.1788 | 21 | 0.0993 | 22 |
| Shanxi | 0.1424 | 28 | 0.1625 | 19 | 0.1749 | 17 | 0.1872 | 19 | 0.1160 | 18 |
| Inner Mongolia | 0.2891 | 11 | 0.2086 | 12 | 0.1824 | 14 | 0.2269 | 15 | 0.1981 | 9 |
| Liaoning | 0.2025 | 16 | 0.1903 | 13 | 0.2351 | 10 | 0.3201 | 9 | 0.1854 | 10 |
| Jilin | 0.1487 | 25 | 0.5324 | 1 | 0.1935 | 11 | 0.2625 | 11 | 0.0912 | 25 |
| Heilongjiang | 0.1518 | 23 | 0.1351 | 23 | 0.1229 | 25 | 0.1638 | 22 | 0.0948 | 24 |
| Shanghai | 0.6819 | 1 | 0.5283 | 2 | 0.7630 | 1 | 0.7220 | 1 | 0.8018 | 1 |
| Jiangsu | 0.5363 | 3 | 0.3976 | 6 | 0.3707 | 6 | 0.4011 | 6 | 0.2586 | 5 |
| Zhejiang | 0.3773 | 8 | 0.3089 | 8 | 0.3108 | 7 | 0.3683 | 8 | 0.2759 | 4 |
| Anhui | 0.2335 | 12 | 0.1898 | 14 | 0.1891 | 12 | 0.2608 | 12 | 0.1634 | 12 |
| Fujian | 0.3529 | 9 | 0.2608 | 9 | 0.2373 | 9 | 0.3131 | 10 | 0.1777 | 11 |
| Jiangxi | 0.1923 | 19 | 0.1748 | 18 | 0.1666 | 18 | 0.2259 | 16 | 0.1563 | 13 |
| Shandong | 0.2275 | 13 | 0.1858 | 15 | 0.1841 | 13 | 0.2222 | 17 | 0.1344 | 15 |
| Henan | 0.1605 | 22 | 0.1455 | 22 | 0.1408 | 21 | 0.1567 | 25 | 0.1120 | 19 |
| Hubei | 0.1960 | 17 | 0.1756 | 17 | 0.1780 | 15 | 0.2292 | 14 | 0.1289 | 16 |
| Hunan | 0.2044 | 15 | 0.1779 | 16 | 0.1753 | 16 | 0.2310 | 13 | 0.1435 | 14 |
| Guangdong | 0.4712 | 4 | 0.4043 | 5 | 0.4242 | 4 | 0.5253 | 3 | 0.3593 | 2 |
| Guangxi | 0.1506 | 24 | 0.1258 | 27 | 0.1273 | 23 | 0.1600 | 24 | 0.0903 | 26 |
| Hainan | 0.3304 | 10 | 0.2387 | 11 | 0.3913 | 5 | 0.4389 | 5 | 0.2359 | 6 |
| Chongqing | 0.4394 | 5 | 0.4405 | 3 | 0.4487 | 3 | 0.4903 | 4 | 0.2338 | 7 |
| Sichuan | 0.2044 | 14 | 0.1510 | 21 | 0.1489 | 20 | 0.1867 | 20 | 0.1162 | 17 |
| Guizhou | 0.1473 | 26 | 0.1126 | 28 | 0.1136 | 26 | 0.1451 | 26 | 0.0889 | 27 |
| Yunnan | 0.1673 | 21 | 0.1262 | 26 | 0.1326 | 22 | 0.1613 | 23 | 0.1114 | 20 |
| Shaanxi | 0.1956 | 18 | 0.1548 | 20 | 0.1548 | 19 | 0.1892 | 18 | 0.1007 | 21 |
| Gansu | 0.1762 | 20 | 0.1344 | 24 | 0.0864 | 30 | 0.1051 | 30 | 0.0678 | 30 |
| Qinghai | 0.0931 | 30 | 0.0871 | 30 | 0.0942 | 29 | 0.1164 | 29 | 0.0814 | 29 |
| Ningxia | 0.3775 | 7 | 0.2432 | 10 | 0.1031 | 27 | 0.1366 | 27 | 0.0850 | 28 |
| Xinjiang | 0.1131 | 29 | 0.0941 | 29 | 0.1024 | 28 | 0.1165 | 28 | 0.0958 | 23 |
