# Supplementary material for: Measurement and spatiotemporal analysis of high-quality development of China’s industry
Source: PLoS One. 2021 Dec 31;16(12):e0259845. doi: 10.1371/journal.pone.0259845 (PMC8719736; doi:10.1371/journal.pone.0259845)
Supplement: Attachment — Submitted filename: 1. Response to Reviewers.docx [file pone.0259845.s002.docx]

**A Reply for Comments and Suggestions of the Manuscript ID (PONE-D-21-12827)**

Dear Editors and reviewers,

We thank the reviewers and editors again for giving very helpful suggestions and comments to improve our manuscript. Our revisions have been made according to the questions and suggestions from two reviewers. The detailed revisions are as follows:

***Questions and suggestions from Reviewer 1:***

The authors have addressed the point of my concern. I am happy with their corrections. Hence, I would like to recommend this manuscript to be published.

[R] Thanks for your help and approval.

***Questions*** ***and Suggestions from Reviewer 2***

[QS1] Despite the authors' explanations, the reviewer is not yet convinced that the reliability and validity of the proposed hybrid method has been well investigated. Please check the similar articles that have been published in Plos One

https://doi.org/10.1371/journal.pone.0228746

https://doi.org/10.1371/journal.pone.0177578

https://doi.org/10.1371/journal.pone.0201604

[A1] We are grateful for the references provided by reviewer. We checked the articles the reviewer recommended. Learning from the research of Dehdasht et al. and the others, we tested the robustness of the combination method proposed in this paper through a sensitivity analysis based on index weights. Because there are too many indicators involved in this study, we first changed the weights of primary indices, then we reflected the results into tertiary indices with equal proportions, and finally we calculated the weights of tertiary indices contained in other primary indices. For the six primary indices, we set three degrees for their changes of weighs and conducted 18 tests. The results confirmed that the method used in this study can evaluate the high-quality development of industry reliably.

[QS2] The conclusion is just listing experimental results. You should provide a clear scientific justification for your work in this section.

[A2] We conducted a sensitivity analysis to justify the robustness of our method and then the rationality of our conclusion.

We promise that all authors agree to the final version, and the authors' names and affiliations are correct, and that funding sources are correctly acknowledged.

Thank you again for your suggestions. Please feel free to contact us if you have any questions.

Best regards,

Yuexiang Yang, Lei Ren, Zhihui Du, Guanqun Tong
